# *Falciparum* Malaria as an Emerging Cause of Fever in Adults Living in Gabon, Central Africa

**DOI:** 10.1155/2014/351281

**Published:** 2014-04-30

**Authors:** Marielle K. Bouyou-Akotet, Christelle L. Offouga, Denise P. Mawili-Mboumba, Laurence Essola, Blondel Madoungou, Maryvonne Kombila

**Affiliations:** ^1^Department of Parasitology Mycology, Faculty of Medicine, Université des Sciences de la Santé, BP 4009 Libreville, Gabon; ^2^Malaria Clinical and Operational Research Unit, Regional Hospital of Melen, Libreville, Gabon; ^3^Emergency Unit, Centre Hospitalier de Libreville, Libreville, Gabon

## Abstract

Following the observed increase of malaria prevalence among older children in Gabon, a descriptive observational study was carried out in 2012 to determine the prevalence of malaria in adults presenting with fever in two health centres of Libreville, the capital city of Gabon. Thick- and thin-blood smears for malaria diagnosis were performed in febrile individuals aged more than 15 years old. Age, use of bed nets, previous antimalarial drug treatment, clinical symptoms, chest radiography results, and available haemoglobin data were also recorded. Among the 304 patients screened, the global malaria frequency was of 42.1% (*n* = 128/34). *Plasmodium (P). falciparum* was the only species identified. The proportion of patients with a clinical malaria requiring parenteral treatment was 38.5%, whereas 47.5% of outpatients had uncomplicated malaria. According to WHO classification, 14 (19.7%) infected patients had severe malaria; neurological and respiratory symptoms tended to be more frequent in case of *P. falciparum* infection. Anaemia was found in 51.5% adults and none had severe anaemia. Almost half of adults consulting for fever in two health centres of the urban city of Libreville have malaria. The use of insecticide-treated bed nets, the screening, and the treatment of individuals with *P. falciparum* microscopic and submicroscopic asymptomatic infection or clinical malaria should be emphasized to reduce the transmission.

## 1. Introduction

In areas with a high level of malaria transmission, children under five years of age and pregnant women represent the most vulnerable population and the primary target of prevention strategies. Reported malaria cases in African adults are scarce. The risk of malaria attacks in residents of malaria-endemic areas decreases with age, because of sequentially acquired immunity which leads to a reduction of life-threatening disease, incidence of mild malaria among adults, and parasite prevalence [1, 2]. However, recent data suggest that malaria burden may be much more frequent than described in nonpregnant adults [3–5].

After the decline in the proportion of malaria cases in Gabonese children between 2005 and 2008, a rebound in infection rates in rural and urban areas occurred [6]. Malaria risk increased over time among older children and decreased among those aged less than five years who benefitted from free preventive measures and treatment over the last seven years [6, 7]. Results have also suggested that in Libreville, malaria transmission remains perennial but becomes mesoendemic, with features of urban epidemiology [6, 7].

Aside from entomological and immunological surveys, measurements of malariometric indices in the exposed population are also essentials to characterize the transmission. Unrecognised parasite reservoir such as asymptomatic or submicroscopic carriers, along with undiagnosed infected adults can contribute to the sustainment of malaria transmission [5, 8, 9]. In Gabon, most epidemiological studies have focused on children, pregnant women, and asymptomatic adults [6, 10–12]. However, in some African settings, it has been reported that severe clinical attacks also occur in adults living in areas with stable malaria and that malaria considerably contributes to death in adults [13, 14]. The last report of clinical malaria attacks in Gabonese adults was done more than 20 years ago; it indicated a prevalence of 12.6% [15].

The present study was designed to determine the prevalence of malaria in adults consulting for fever in two hospitals of Gabon, Central Africa, and* P. falciparum *attacks clinical features at admission.

## 2. Patients and Methods

### 2.1. Study Site and Population

This is an observational and descriptive study using data collected during a prospective cross-sectional survey conducted from December 2011 to October 2012 in Gabon, at Libreville, the capital city, and at Melen, a suburban area located 11 km north of Libreville. Patients were recruited at the Centre Hospitalier Universitaire de Libreville (CHL) and the regional hospital of Melen (RHM), two sentinel sites for malaria surveys selected by the Malaria National Control Program (MNCP). In sentinel sites, febrile patients are routinely screened for malaria infection; therefore, data collection was part of the normal activities at the CHL and at the RHM. As malaria transmission is perennial without significant fluctuation throughout the year, the study team collected data over at least one rainy and one dry season in each site.

Febrile outpatients and inpatients, aged more than 15 years, were included after they accepted to participate in the study. Oral informed consent was obtained to complete the demographic and medical history sections of a case report form (CRF). Tympanic temperature, history and duration of fever, age, sex, bed net use, prior self-treatment with an antimalarial drug, and clinical symptoms leading to the consultation were recorded into the CRF. In order to minimize the frequency of coincidental parasitaemia in case of comorbidity, patients with known chronic infection such as HIV, hepatitis, and tuberculosis were not included.

### 2.2. Malaria Diagnosis

For each patient, matched thick- and thin-blood smears were prepared and stained with 20% Giemsa. Thick smears were screened for the presence of malaria parasites according to the Lambaréné method [16]. Carefully, 10 *μ*L of blood was layed on a 10 by 18 mm area of a microscope slide and then dried and stained. Parasitemia was expressed as the number of parasites per microlitre of blood (p/*μ*L), and parasite species were identified in the matched thin-blood smears. Smears were read using a light microscope (×100 oil immersion lenses). Quality control of the blood smears was performed: a second microscopist, blinded to the results of the first reading, read the slides. In case of disagreement, slides were controlled by a third microscopist and the mean of the two closest readings of parasitaemia was taken. A patient was considered negative if no parasite was observed after the examination of at least 100 oil immersion fields in a thick-blood smear.

### 2.3. Haematology

Available haemoglobin (Hb) measurements were used for the analysis of anaemia. According to the WHO classification, anaemia was defined as a Hb concentration below 11 g/dL and classified as severe (Hb < 5 g/dL), moderate (5 ≥ Hb < 8 g/dL), or low (8 ≥ Hb < 11 g/dL) type.

### 2.4. Definitions

According to WHO 2010 and Newton and Krishna classification of African population modes of disease presentation, malaria patients were classified into three groups: (1) severe malaria patients who strictly met the WHO 2000 criteria for severe malaria; (2) moderate malaria patients who did not have any clinical or biological features of SM according to WHO classification but required parenteral treatment and hospitalization because of symptoms such as asthenia, vomiting, convulsions; (3) uncomplicated malaria patients, that were, febrile outpatients who did not have any features of severe or moderate disease and where able to tolerate oral medication [17, 18].

Fever was defined as a tympanic temperature > 37.5°C, dyspnoea as an observed respiratory rate ≥ 20 beats/minutes. Level of consciousness was determined using the Glasgow coma score and impaired consciousness as a GCS < 10. Any degree of impairment of consciousness or any other neurological involvement was considered as a potentially serious neurological manifestation. Chest radiography results were used for the diagnosis of pulmonary infection.

### 2.5. Ethical Consideration

The study aims were clearly explained to the ward staffs. Patients were informed about the protocol, and their oral consent was required prior to data collection. Although it was a noninvasive study, this oral consent was sought to obtain medical history and to use the demographic, clinical and biological data. Data were kept confidential. The study was approved by the Ministry of Health. As a reference laboratory for the MNCP, the Department of Parasitology Mycology (DPM) has health authorities' approval to monitor the evolution of malaria morbidity throughout the entire country through sentinel sites. Malaria diagnosis was free of charge and infected patients were treated according to the national policy. For patients aged between 15 and 20 years old, individual oral assent and oral consent of legal guardian were required prior to their inclusion.

### 2.6. Sample Size Calculation

According to WHO sample size tables for prevalence studies, the sample size calculation was based on 5% precision with 95% level of confidence and an expected prevalence of symptomatic malaria of 15% [19]. This expected prevalence was based on the assumption that the prevalence of symptomatic adults will be at least comparable to the reported prevalence of asymptomatic malaria in the country [11, 12]. Therefore a minimum of 196 screened adults was necessary.

### 2.7. Statistical Analysis

All data were entered and cleaned using Epi-info version 3.3.2 (2005 CDC Atlanta) and analyzed with Stata. Qualitative variables were summarised as frequencies and percentages. Median with interquartile ranges (percentiles 25th and 75th) were used for continuous variables such as age, Hb, and parasitaemia. The results were compared with the Chi-square or Fisher's exact test or the nonparametric tests (Mann Whitney and Kruskal Wallis) as appropriate. Bivariate analysis was used to determine possible factors that could be associated with malaria. A multivariate analysis was then performed using a forward stepwise likelihood ratio logistic regression, and considering the presence of malaria parasite (Yes/No) as the dependent variable, and variables which resulted in a *P* value of <0.20 in the bivariate analysis as the independent variables for this analysis. The significance level of all comparisons was set at a *P* value < 0.05.

## 3. Results

During the study period, 1654 febrile patients were screened for malaria by the study team, only 361 (22%) were adults. Data from 304 were available and analysed. One hundred twenty patients were referred by the outpatient unit, and 184 came from the emergency ward. The median age of the patients was 25 [19–34] years, and 29% (*n* = 88) were male. The majority of the patients attended the hospital after two days of illness duration (2 [2–5] days), and 15.3% (*n* = 30/196) of them had taken an antimalarial treatment before the consultation (Table 1). Artemisinin-based combination therapies (ACTs) represented 99% of the used drugs for previous self-medication; only one patient had taken tablets of quinine before the consultation. The median temperature was 39.0 [38.5–39.0]°C. Haemoglobin values that were available for 204 patients, ranged between 5.6 g/dL and 15.3 g/dL. Anaemia was detected in more than half of them and 18 (8.8%) had moderate anaemia. Bed net use was infrequent (Table 1).

### 3.1. Malaria Infection

A total of 128 (42.1%) among the 304 adults had a positive blood smear, with only three harbouring gametocytes with asexual forms.* P. falciparum *was the only species identified. Parasite densities varied from 28 to 214775 p/*μ*L, the median value being 5600 [869–23310] p/µL. Thirty-six (28.1%) out of the 128 infected patients had a parasite count below 1000 p/*μ*L, 61 (47.7%) had more than 5,000 p/*μ*L, and five (3.9%) had a count greater than 100,000 p/*μ*L. The* P. falciparum *infection rate did not vary significantly with bed net use or previous antimalarial treatment (Table 1). Malaria-positive and uninfected groups were almost indistinguishable in terms of the time between the onset of symptoms and presentation at hospital, with a median of two days. Infected patients reported maximum fever duration of six days, whereas fever was present up to a maximum of 23 days before consultation in the group of malaria-negative patients. The proportion of patients with malaria infection requiring parenteral treatment (moderate or severe malaria) was 38.6% (*n* = 71/184 inpatients), whereas 47.5% (*n* = 57/120 outpatients) had uncomplicated malaria (*P* = 0.1).* P. falciparum *prevalence did not correlate with age: it was 44.8% in patients aged less than 31 years, 34.6% among those age between 31 and 45 years, and 43.5% in those over 45 years.

### 3.2. Symptoms Associated with Malaria

Among the malaria patients, 77.3% (*n* = 99/128) did not present clinical and radiological signs suggestive of other infection. For outpatients, the main reasons for consultation, except fever or history of fever, were fatigue and headache. In the hospitalized group, fatigue, digestive symptoms, pallor, and respiratory symptoms (more than 25% of patients) predominated (Table 2). Aside from fever, a total of 26 (36.6%) of them had multiple symptoms; the predominant association was represented by the vomiting plus pallor and fatigue (21.1%). The majority of the infected inpatients (*n* = 57/71) had moderate malaria. Clinical signs of severe malaria were present in 14 (19.7%)* P. falciparum *infected inpatients: six (42.9%) had neurological forms (OR 5.1 95%CI 1.0–26.1), three (21.4%) had respiratory distress syndrome, and five (35.7%) had repeated vomiting (Table 2).

After the multivariate analysis, none of the recorded clinical symptoms was significantly associated with malaria (Table 2).

### 3.3. Anaemia

Haemoglobin levels were analysed in the subgroup of 204 patients with available data. The majority of anaemic patients (*n* = 87/105; 82.9%) had low anaemia. Hb levels and anaemia prevalence were not related to the presence of malaria parasites: 44 among 83 (53.0%) infected patients and 55 out of 121 (45.4%) had a Hb level below 11 g/dL. No case of severe malarial anaemia (Hb below 5 g/dL with positive blood smear) was observed. Five malaria patients had a Hb level below 7.0 g/dL, whereas the lowest Hb value was 7.1 g/dL among the uninfected patients.

## 4. Discussion

Since the adoption and implementation of ACTs and insecticide-treated nets (ITNs), efforts have been made to obtain data on population-based prevalence and transmission of malaria in several malaria-endemic countries. These informations should help to identify priority areas for evaluation, monitoring, and improvement of implemented disease control strategies. Epidemiological studies generally focus on children, pregnant women, and asymptomatic individuals [7, 10, 20, 21]. However, following the decrease in global malaria prevalence observed over the past few years, the WHO recommends universal ITN coverage to further reduce malaria transmission. Recent data on the burden of malaria in adults from areas with perennial transmission are lacking. These adults are considered to be semi-immune and would represent an important parasite reservoir because they are frequently asymptomatic or submicroscopic parasite carriers [9, 22, 23]. Moreover, the epidemiology of* P. falciparum *is changing through urbanization and implementation of interventions [24, 25]. This decline could have an impact on the level of acquired specific immunity; thus, the clinical and epidemiological features of malaria infection could be modified according to age [26, 27].

Compared to the reported prevalence among children aged less than five years old (17.9%), a surprisingly high microscopic prevalence of malaria was found among febrile adults living in the capital city of Gabon, Central Africa [6]. This prevalence is threefold higher than those reported 20 years ago (42.1% versus19.0%) and three- to seven fold higher than that of asymptomatic adults living in different areas of Gabon [11, 12, 15]. Recent reports have already highlighted the greatest frequency of malaria in febrile older children in different areas of the country [6, 7]. A persistent high number of malaria cases in older children and in adults were observed in Zimbabwe, following the scale up of disease control strategies [5]. Therefore, the population who did not benefit for free malaria interventions (i.e., children older than 5 years old and nonpregnant adults) now carries the highest burden of infection. Our results support the notion of a changing malaria epidemiology profile in Libreville. Indeed, changes in malaria transmission are preceded with a shift of malaria burden according to age [21]. The epidemiology of malaria is also modified through urbanisation and implementation of interventions [26–28]. For example, in Thies, an urban area of Senegal with seasonal mesoendemic malaria, the most affected population was aged 4 to 20 years [29]. Libreville is actually undergoing unplanned urbanisation, which connotes proximity to mosquito breeding grounds. In Uganda, it was reported that malaria risk in adults is associated with this proximity and with the absence of bed net use [30].

A low frequency of parasite density (PD) below 1000 p/*μ*L was also noticed. The median PD was 5600 p/*μ*L, higher than the mean PD observed in adult hospitalized patients at the CHL in 1991 (1000 p/*μ*L) and the PD in asymptomatic patients from Lambaréné (<200 p/*μ*L) [11, 15].

Identifying clinical factors associated with malaria can help to conclude to an incidental parasitaemia or to provide appropriate management of febrile infected patients, at least to identify those who require intensive care unit admission. Gabonese children with complicated malaria present more frequently prostration, seizures, and pallor; the predominant signs, presented by the parasitaemic hospitalized adults, were also found in a similar frequency among uninfected individuals [31]. Altered level of consciousness and seizures were the main neurological symptoms in the case of a positive blood smear as reported in Tanzanian adults [32]. The observed absence of significant clinical risk factors could be explained by a coincidental* P. falciparum *in patients with a fever due to other infections. However, it was recently reported that intensifying malaria control modifies the threshold of parasitaemia to be used to define* P. falciparum *attacks, that is, fever attributable to malaria; pyrogenic threshold levels of 1000 p/*μ*L for adults and of 5000 p/*μ*L in children for attributing fever episodes to* P. falciparum* malaria were defined in an endemic area of Senegal [26]. Thus, the high prevalence and the high parasite densities observed here indicate malaria as the leading cause of the fever, although clinical symptoms of patients remain unspecific. Therefore, there is actually a substantial proportion of febrile adults (38%) who had a malaria attack classified as either moderate or severe according to WHO and Newton and Krishna classifications [17, 18]. Nineteen percent of hospitalized patients had WHO-defined severe* falciparum* malaria. A higher frequency was reported in adults from Asia and non-malaria-endemic settings [33, 34]. Studies performed the last 20 years confirm the lowest prevalence of SM in African population who already experienced malaria episodes [33–35].

Although this was not a study designed to analyze the risk factors for severe malaria in adults, most of the clinical symptoms presented by the patients were not found to be associated with the presence of* P. falciparum* like recently reported by Newton et al. [36]. However, neurological symptoms and respiratory distress that are recognized as predictive of deaths in adults were also more prevalent in case of positive blood smear [36, 37].

More than half (51.5%) of the patients had anaemia, which was not significantly related to the presence of malaria parasites. The majority of anaemic individuals had low anaemia like that reported in other settings [34]. According to WHO, a severe public health problem is a disease with a prevalence greater than 40% in any group. Therefore, evaluating the real burden of anaemia in adult Gabonese population is needed to determine strategies for management and prevention after eliminating known risk factors such as HIV infection, tuberculosis, and arboviruses which are also frequent in adults malaria patients [38, 39]. Nutritional deficiencies and malaria are the main contributors of low Hb levels in African children; further studies on adults are required. Additionally, submicroscopic parasitaemia and multiplicity of infections that are known to be associated with anaemia were also often observed in adults living in Libreville [40, 41].

This study has some limitations. First, it is health-facility-based study and it did not include patients within the community. It is important to underline that, in the case of fever, a majority of people aged more than 15 years directly obtain an antimalarial drug from a pharmacist; also some patients could have been missed by the study team although they had a daily presence at hospital. Therefore, the burden of malaria could be overestimated or underestimated. A second limitation could be the absence of molecular diagnosis of malaria, which is essential for the real estimation of malaria burden [8].

## 5. Conclusion

This descriptive study highlights the emergence of malaria in febrile adults living in an urban city of Gabon. Malaria attacks which require parenteral treatment are now more frequently described in adult patients, with a prevalence of severe malaria exceeding 10%. Whether this increased prevalence is due to high exposure or to an inadequate acquired immunity remains an important issue. Nevertheless, this population may contribute to the recent rebound of malaria observed in the country as an important parasite reservoir. Additionally, the present data highlight a challenge to control and eliminating efforts in the country. Adults with any evidence of* P. falciparum *infection should be promptly treated with antimalarials before further investigation on other possible causes. The use of ITNs, screening and treatment of individuals with microscopic or submicroscopic asymptomatic infection or clinical malaria should be emphasized and could reduce the transmission of malaria in the country.

## Figures and Tables

**Table 1:** Patient characteristics.

Continuous variables	All (*N* = 304)	Malaria-positive (*n* = 128)	Uninfected (*n* = 176)	*P*
Age, years*	25 (19–34)	24 (19–33)	26 ( 20–2 )	0.37
Duration of illness, days*	2 (2–4)	2 (2–5)	2 (2-2)	0.88
Haemoglobin, g/dL*	10.8 (10.1–11.8)	11.0 (9.9–11.7)	10.8 (10.1–11.8)	0.14
Outpatients, *n* (%)	120 (39.5)	57 (44.5)	63 (35.8)	0.12
Inpatients, *n *(%)	184 (60.5)	71 (55.5)	113 (64.2)	0.12
Bed net use, *n* (%)	53 (17.4)	23 (17.90)	30 (17.1)	0.83
Previous self-treatment with antimalarial, *n* (%)**	30 (15.3)	9 (12.7)	21 (16.8)	0.27
Anaemia, *n* (%)***	105 (51.5)	39 (54.5)	66 (47.0)	0.19

*Median (IQR). **Data available for 196 patients. ***Data available for 204 patients.

**Table 2:** Clinical symptoms presented by hospitalized patients (inpatients) according to the presence of *P. falciparum* infection.

	All (n = 184)		Malaria patients (n = 71)		Uninfected patients (*n* = 113)		Univariate analysis OR [95% CI]	P	Multivariate analysis aOR [95% CI]	P
	*N*	%	*N*	%	*N*	%				
Fatigue	30	16.3	22	31.0	8	7.1	0.6 (0.2–1.4)	0.2	0.4 (0.2–1.1)	0.09
Arthralgia	31	16.8	11	15.5	20	17.7	0.9 (0.4–2.1)	0.9	0.7 (0.3–1.9)	0.56
Pallor	59	32.1	24	33.8	35	31.0	1.3 (0.7–2.4)	0.3	1.2 (0.9–2.2)	0.08
Vomiting	64	34.8	19	26.8	45	39.8	0.6 (0.3–1.2)	0.2	0.5 (0.2–1.2)	0.12
Diarrhea	38	20.6	10	14.1	28	24.8	0.5 (0.3–1.2 )	0.2	0.8 (0.3–1.9)	0.57
Abdominal pain	17	9.2	3	4.2	14	12.4	0.3 (0.1–1.2)	0.1	0.3 (0.1–1.3)	0.12
Deep breathing	4	2.2	3	4.4	1	0.1	5.4 (0.6–52.7)	0.1	3.4 (0.3–35.7)	0.29
Cough	53	28.8	20	28.2	33	29.2	0.9 (0.3–1.8)	0.6	0.8 (0.3–1.8)	0.61
Pneumonia syndrome	50	27.2	19	26.8	31	27.4	0.9 (0.5–2.0)	0.8	0.8 (0.3–2.1)	0.58
Seizures	5	2.7	3	4.2	2	1.8	2.7 (0.4–16.4)	0.3	2.7 (0.4–19.2)	0.33
Impaired consciousness	3	1.6	3	4.2	0	0.0	NA		NA	
